# Intraoperative indocyanine green video angiography (ICG–VA) with FLOW 800 software in complex intracranial aneurysm surgery

**DOI:** 10.1186/s41016-021-00247-z

**Published:** 2021-06-01

**Authors:** Tao Xue, Ruming Deng, Bixi Gao, Zilan Wang, Chao Ma, Wanchun You, Yun Zhu, Zhouqing Chen, Zhong Wang

**Affiliations:** 1 Department of Neurosurgery & Brain and Nerve Research Laboratory, The First Affiliated Hospital of Soochow University, 188 Shizi Street, Suzhou, 215006 Jiangsu Province China; 2 Department of Neurosurgery, Bozhou People’s Hospital, Bozhou, Anhui Province China

**Keywords:** Aneurysm, Bypass, Clipping, FLOW 800, ICG-VA

## Abstract

**Background:**

Indocyanine green video angiography (ICG–VA) is a safe and effective instrument to assess changes in cerebral blood flow during cerebrovascular surgery. After ICG-VA, FLOW 800 provides a color-coded map to directly observe the dynamic distribution of blood flow and to calculate semiquantitative blood flow parameters later. The purpose of our study is to assess whether FLOW 800 is useful for surgery of complex intracranial aneurysms and to provide reliable evidence for intraoperative decision-making.

**Methods:**

We retrospectively reviewed patients with complex aneurysms that underwent microsurgical and intraoperative evaluation of ICG-VA and FLOW 800 color-coded maps from February 2019 to May 2020. FLOW 800 data were correlated with patient characteristics, clinical outcomes, and intraoperative decision-making.

**Results:**

The study included 32 patients with 42 complex aneurysms. All patients underwent ICG-VA FLOW 800 data provided semiquantitative data regarding localization, flow status in major feeding arteries; color maps confirmed relative adequate flow in parent, branching, and bypass vessels.

**Conclusions:**

FLOW 800 is a useful supplement to ICG-VA for intraoperative cerebral blood flow assessment. ICG-VA and FLOW 800 can help to determine the blood flow status of the parent artery after aneurysm clipping and the bypass vessels after aneurysm bypass surgery.

**Supplementary Information:**

The online version contains supplementary material available at 10.1186/s41016-021-00247-z.

## Background

Complex cerebral aneurysms contain not only giant aneurysms (greater than 25 mm in diameter) but also some aneurysms characterized by large (15–25 mm), difficult locations, previous treatment, presence or absence of collateral circulation, intraluminal thrombus, and calcification of the aneurysmal wall [1]. To discuss the selection of endovascular or surgical treatment for complex intracranial aneurysms is beyond the aim of this article. However, the treatment method should be decided carefully with the consideration of advanced endovascular technology such as modified stents and novel embolic agents perhaps improving therapeutic effect for these complex aneurysms [2]. Once the surgical procedure is adopted, direct clipping becomes the most common strategy [3]. When the direct approach is impractical or at-risk, alternative approaches including trapping with or without flow replacement (bypass), primary proximal vessel occlusion (Hunterian ligation), and distal vessel occlusion can be used [4]. The complexity of these aneurysms often makes the operation more difficult, and they are accompanied by a high occurrence of complications. In consequence, some intraoperative assistant equipment is required to avoid such lesions.

Indocyanine green video angiography (ICG-VA) has been widely used in vascular neurosurgery, especially in cases of aneurysms, arteriovenous malformations (AVMs), and bypass, to assess the cerebral blood flow since its introduction in 2003 [5–9]. The potential disadvantages of conventional ICG-VA include that it only evaluates the blood flow within the field of view and lacks quantitative analysis of the angiography images [10].

FLOW 800 (Carl Zeiss, Oberkochen, Germany), a new surgical microscope-integrated software program, allows ICG-VA data to be analyzed in a semiquantitative way by creating a color-coded map [11]. Surgeons can comparatively evaluate the flow rate at user-defined regions of interest (ROIs) within the operative field by the analysis of fluorescence profiles [11]. The application of FLOW 800 for microsurgical treatment of AVMs, regular intracranial aneurysms, and moyamoya disease (MMD) has been reported in some research [11–15], and a case report including 3 patients of complex intracranial aneurysms can be obtained in the available literature [16]. However, large studies for complex intracranial aneurysms are rare. The purpose of this study was to present our single-center experience with the application of intraoperative ICG-VA and hemodynamic analysis with FLOW 800 in complex intracranial aneurysms.

## Methods

This is a single-center, retrospective study of consecutive patients that underwent microsurgical operations of complex intracranial aneurysms and intraoperative assessment of ICG-VA and FLOW 800 color-coded map from February 2019 to May 2020. The exclusion criteria include (a) patients who were treated via endovascular methods alone, (b) patients allergic to ICG, (c) pediatric patients, (d) patients who had surgical treatment where FLOW 800 technology was not used, and (e) not complex aneurysm. Based on the institutional guidelines, the ethics committee approval was not required for our study.

### Procedures details

Before surgery, the patients underwent a routine computed tomography angiography (CTA) or/and digital subtraction angiography (DSA) scan to confirm aneurysm size, location, and morphology. The aneurysm treatment decision was made through a collaboration of vascular neurosurgeons and neuro-interventionalists. Patients were considered for bypass if standard microsurgical clipping was not feasible. Surgical approaches, clippings, and bypasses were all performed in the standard way by the senior author. For approaches, a pterional or lateral supraorbital craniotomy was for aneurysms of the internal carotid artery (ICA), posterior communicating artery (PcomA), the middle cerebral artery (MCA), and the basilar artery (BA). Anterior communicating artery (AcomA) aneurysms were exposed via pterional, lateral supraorbital, or a midline approach. The anterior inferior cerebellar artery (AICA) and posterior inferior cerebellar artery (PICA) aneurysms were clipped by a suboccipital approach. The posterior cerebral artery (PCA) and the superior cerebellar artery (SCA) aneurysms were clipped after subtemporal craniotomy. For clipping, we carefully dissected the arachnoid membrane to achieve the proximal control of the main arteries until a temporary clip was able to be placed. After the aneurysm neck and dome were exposed completely, the surgical findings were consistent with the preoperative angiography to consider a clipping strategy and to select appropriate aneurysm clips. For bypass, ICA/external carotid artery (ECA)-radial artery (RA)-second/third segment of the MCA (M2/M3) bypass, ECA-RA-PCA, or superficial temporal artery (STA)-M2/M3/M4 bypass were usually performed to compensate for the sacrificed blood flow when the aneurysm was isolated or to improve the existing symptoms of cerebral ischemia. Anastomoses were performed under anesthetic burst suppression and with the patient’s systolic blood pressure 10 to 20 mmHg higher than the baseline during temporary occlusion. For intraoperative assessment of aneurysm occlusion, parent, and bypass artery patency, ICG-VA with/without microdoppler ultrasound was performed. After surgery, some patients were admitted to an intensive care unit on basis of individual situation. Routine cranial computed tomography (CT) scans were performed to exclude rebleeding immediately after recovery from general anesthesia and cerebral infarction on the first postoperative day. Transcranial doppler (TCD) ultrasound was performed daily in patients with SAH to monitor the blood flow velocity. Cerebral vasospasm occurred when a mean blood flow velocity ≥ 120 cm/s and/or an augment by ≥ 50 cm/s within 24 h [17]. For patients with possible cerebral vasospasm or other neurological deficits, an urgent cranial CT scan with angiography and perfusion was performed additionally.

### Fluorescence angiography and FLOW 800 analysis

An OPMI Pentero surgical microscope with integrated ICG technology (Carl Zeiss GmbH, Oberkochen, Germany) was used for intraoperative analysis of cerebral blood flow. The application of ICG for microsurgery was proven technique and has been described previously in detail [18]. After 25 mg of ICG was administered intravenously, fluorescence could be induced by the integrated light source (about 800 nm in wavelength) and filmed by integrated camera as INFRARED 800 (IR 800) video which enabled a real-time assessment of arterial, cortical, and venous blood flow under visual field. The times of ICG-VA runs was decided by neurosurgeon. In general, intraoperative ICG-VA was performed twice at baseline and after aneurysm treatment.

The FLOW 800 analysis software (Release 2.21, Carl Zeiss GmbH, Oberkochen, Germany) was also integrated with the surgical microscope, allowing for real-time and semi-quantitative analysis of the cerebral blood flow via IR 800 video. An intuitive scale highlights early appearance of fluorescence in red (e.g., arteries), medium appearance in yellow/green (e.g. cortical capillaries), and late appearance in blue (e.g., veins). The color-coded maps generated by FLOW 800 software simply provide clearer indications rather than absolute measurements. To receive detailed fluorescence intensity curve of a specific vessel or area within the field of view, up to 8 definable regions of interest (ROIs) can be selected and analyzed. In our study, ICG-VA and FLOW 800 analysis were performed as clinical indications. The fluorescence curves which have been thoroughly studied by previous studies [14–16] were not analyzed because these analyses take longer, which is not available or practical during surgery. In other word, the fluorescence curves of ROIs can provide hemodynamic analysis of the vessels but we did not use them during our operation and depended only on the FLOW 800 color-coded map that is effective in guiding intraoperative procedures.

### Data collection

The following variables of every patient were collected retrospectively: age, gender, subarachnoid hemorrhage (SAH), Glasgow Coma Scale (GCS), Hunt-Hess grade, modified Rankin Score (mRS), aneurysm number, size, complexity, location, intraoperative findings, FLOW 800 results, postoperative imaging, and complications.

## Results

### Patient and aneurysm characteristics

A total of 32 consecutive patients with 42 complex aneurysms were enrolled in this study. Patients ranged in age from 17 to 77 years (mean age of 54 years) with a gender ratio of 1:5.4 (male/female). Thereof, 16 patients were treated for unruptured aneurysms and 16 patients with SAH were treated for a ruptured aneurysm. Baseline patient characteristics including GCS, Hunt-Hess grade, and mRS are summarized in Table 1.

**Table 1:** Baseline patient and aneurysm characteristics

Variable	Value (*n* = 32)
Age (years)	
Mean ± SD	53.91 ± 14.45
Range	17–77
Gender (%)	
Female	27 (84%)
Male	5 (16%)
SAH (%)	16 (50%)
GCS (%)	
0–3	0 (0%)
4–6	2 (6%)
7–9	2 (6%)
10–12	3 (9%)
13–15	25 (78%)
Hunt-Hess grade (%)	
0 (unruptured)	16 (50%)
1	5 (16%)
2	4 (12%)
3	5 (16%)
4	2 (6%)
5	0 (0%)
mRS (%)	
0	0 (0%)
1	11 (34%)
2	8 (25%)
3	3 (9%)
4	3 (9%)
5	7 (22%)
Aneurysm number	42
Aneurysm size (mm)	
Mean ± SD	16.81 ± 16.25
Range	1.6–74.7
Complexity (one aneurysm may have several complexity)	
Giant (≥ 25 mm)	6
Large (15 mm–25 mm)	6
Difficult locations	14
Presence of collateral circulation	4
Intraluminal thrombus	3
Calcification of wall	3
Previous treatment	2
Others	8
Multiple aneurysms requiring simultaneous management	5 (patients)
With sellar tumor	1
Wide neck	1
Dissecting aneurysm	1
Aneurysm location (%)	
Anterior circulation	32 (76%)
AComA	3 (7%)
MCA	9 (21%)
ICA-PComA	7 (17%)
ICA	13 (31%)
Posterior circulation	10 (24%)
PICA	1 (2%)
AICA	2 (5%)
PCA	2 (5%)
SCA	1 (2%)
BA	4 (10%)

*SD* = standard deviation, *SAH* = subarachnoid hemorrhage, *GCS* = Glasgow Coma Scale, mRS = modified Rankin score, *AVM* = arteriovenous malformation, *AComA* = anterior communicating artery, *MCA* = middle cerebral artery, *ICA* = internal carotid artery, *PComA* = posterior communicating artery, *PICA* = posterior inferior cerebellar artery, *AICA* = anterior inferior cerebellar artery, *PCA* = posterior cerebral artery, *SCA* = superior cerebellar artery, *BA* = basilar artery

The mean aneurysm size was 16.81 ± 16.25 mm, ranged from 1.6 to 74.7 mm. Of 42 aneurysms, 32 (76%) aneurysms were located at the anterior circulation including 3 (7%) at the anterior communicating artery, 9 (21%) at the middle cerebral artery, 7 (17%) at the posterior communicating artery and 13 (31%) at the internal carotid artery; 10 (24%) aneurysms were located at the posterior circulation comprised of 1 (2%) at the posterior inferior cerebellar artery, 2 (5%) at the anterior inferior cerebellar artery, 2 (5%) at the posterior cerebral artery, 1 (2%) at the superior cerebellar artery, and 4 (10%) at the basilar artery. Baseline aneurysm characteristics including the complexity of included aneurysms are listed in Table 1.

### Representative cases

We presented the following three cases to illustrate the values of ICG-VA with FLOW 800 analysis in complex intracranial aneurysm surgery. In addition, the details of the operative information and FLOW 800 findings for individual cases were summarized in Table S1.

### Case 1

**Fig. 1:**
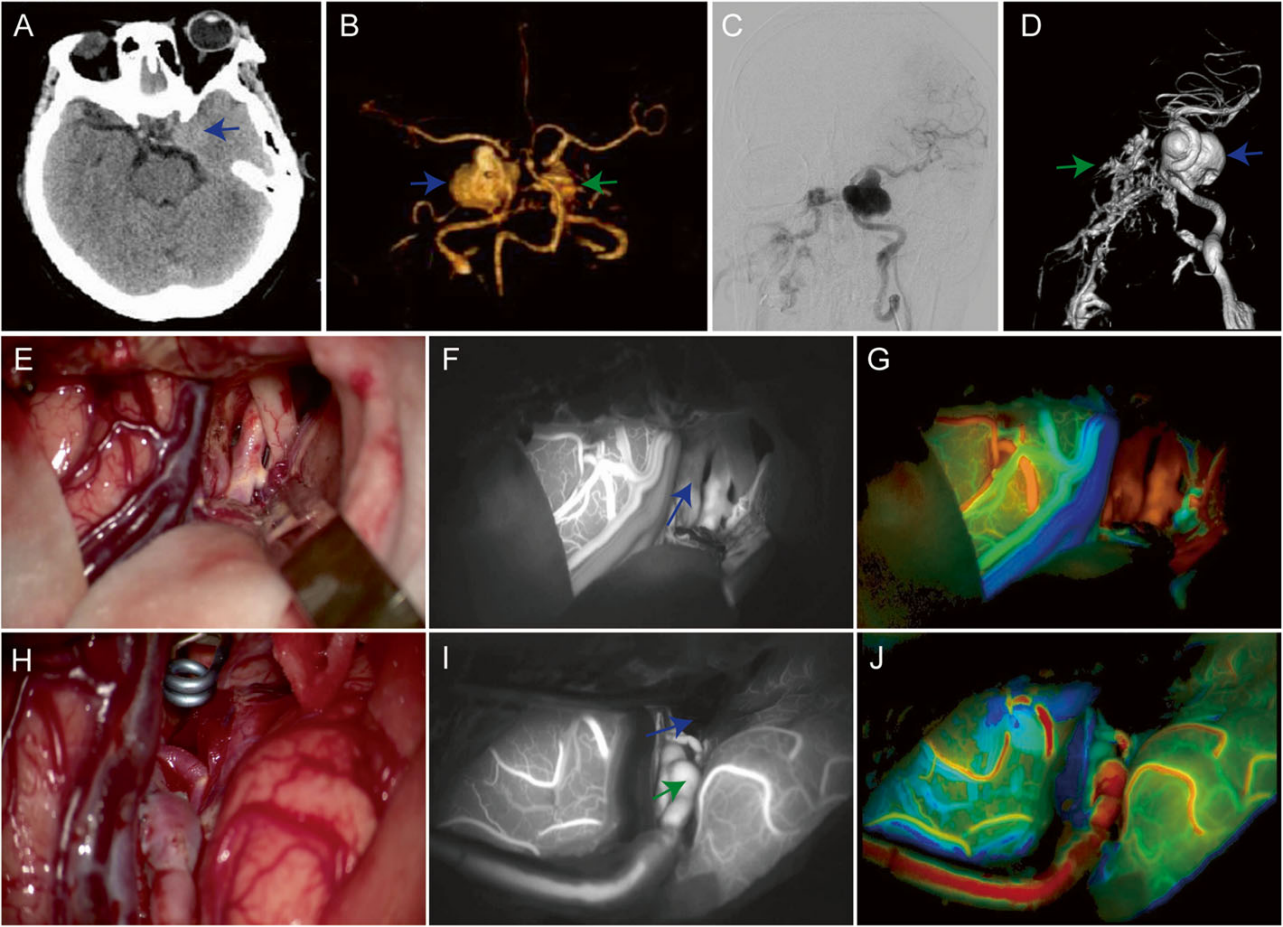
A left internal carotid artery (ICA) C4 segment unruptured giant aneurysm (25 × 20 mm, blue arrow) with carotid-cavernous fistula (CCF, green arrow) was diagnosed by computed tomography (**a**), computed tomography angiography (**b**), digital subtraction angiography (**c**), and 3-dimensional rotational angiography (**d**). During surgery, the aneurysm was exposed (**e**). Then, conventional indocyanine green video angiography (ICG-VA) showed aneurysm (**f,** blue arrow), parent artery, and peripheral blood vessels. FLOW 800 provided a color-coded map that displayed the brain vessels in contrasting colors depending on the fluorescence appearance after ICG injection (arteries: red, cortical capillaries: yellow/green, veins: blue) (**g**). After high flow extracranial-to-intracranial (EC-IC) bypass (middle cerebral artery M3 segment-radial artery-ICA), we isolated aneurysm at ICA initial part and ICA before posterior communicating artery (PComA) branching (**h**). Another ICG-VA (**I**) and FLOW 800 analysis (**j**) showed the aneurysm disappeared (blue arrow) and the bypass vessel was fluent (green arrow)

A 61-year-old female patient (No. 3 in Table S1) presented with pulsatile exophthalmos and headache. Imaging detected one giant aneurysm (25 × 20 mm) with carotid-cavernous fistula (CCF) located on the left ICA (Fig. 1a–d). The left pterional approach was used for craniotomy. After complete microsurgical dissection of the aneurysms, the patient underwent intraoperative ICG–VA and FLOW 800 analysis (Fig. 1e–g). The left forearm was cut straight along the radial artery (RA) which was dissociated, washed with papaverine, and soaked in normal saline for bypass. Then, we took a straight incision in the neck and exposed the left CCA upward to the bifurcations of ICA and ECA. Afterwards, following high flow extracranial-to-intracranial (EC-IC) bypass (M2-RA-ICA), we trapped aneurysm at initial part of ICA and the distal end of the PComA. Intraoperative ICG-VA, FLOW 800 analysis were repeated to confirm the aneurysm disappeared and the bypass vascular was fluent (Fig. 1h–j). In addition, intraoperative ultrasound was applied to reconfirm the anastomotic graft was unobstructed. After operation, there was no hemorrhage in the operative area on CT. The complete obliteration of the aneurysm and the patency of bypass were confirmed by postoperative CTA two days after surgery. The cerebral blood perfusion was also good on CTP (P1 in Fig. S1). At discharge, the patient had signs of injury to the left oculomotor nerve, but the symptoms of eyeball pulsation and headache had basically disappeared.

### Case 2

**Fig. 2:**
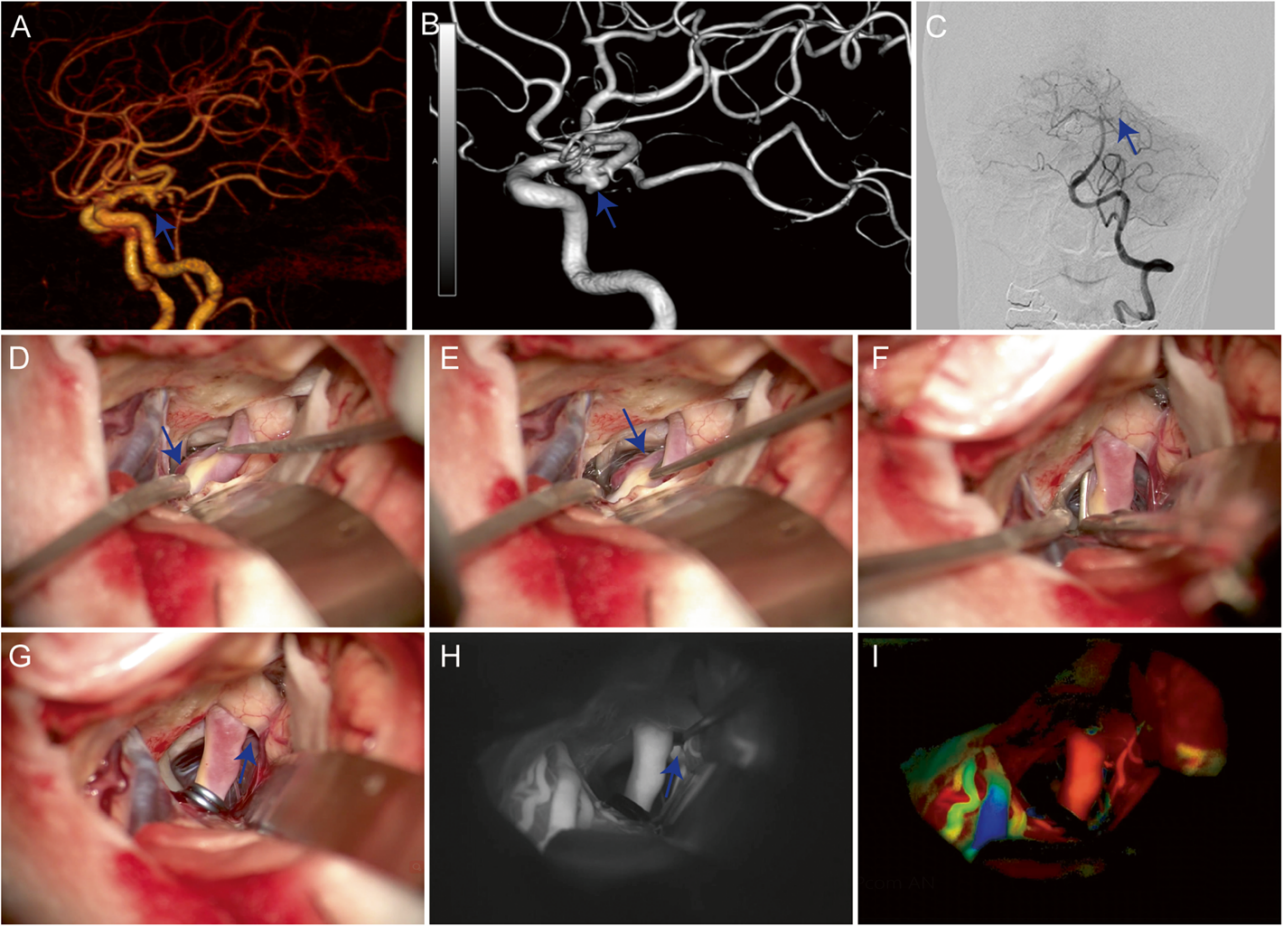
A left posterior communicating artery (PComA) aneurysm (4 × 3 mm, blue arrow) was diagnosed by computed tomography angiography (**a**), digital subtraction angiography (DSA) (**b**). DSA also indicated that the artery (blue arrow) was fetal-type posterior cerebral artery (**c**). During surgery, the lateral (**d**) and body (**e**) of aneurysm was exposed. The aneurysm neck was clipped with a cross-window aneurysm clip (**f**) and PComA (blue arrow) was visible (**g**). Conventional ICG-VA (**h**) and FLOW 800 analysis (**i**) showed clipping was satisfactory and the parent artery was unobstructed (blue arrow)

A 62-year-old male patient (No. 22 in Table S1) was admitted due to an aneurysm of about 4 × 3 mm in size found in the left posterior communicating artery (L-PComA). CTA and DSA indicated that the aneurysm was located outside of the left ICA, pointing outward and downward, with obvious peripheral adhesion. The PComA went out from the proximal aneurysm neck and the ipsilateral PCA was fetal-type which makes it very important to preserve PcomA intraoperatively (Fig. 2a–c). The optic nerve, ICA, PComA, and the lateral of aneurysm were exposed after frontal lobe was dragged by brain spatula. After the surrounding arachnoid was separated carefully, the proximal part of PComA and the body of the aneurysm are visible. We dissociated the aneurysm neck and clipped the aneurysm neck with a cross-window aneurysm clip (Fig. 2d–g). Intraoperative FLOW 800 analysis indicated that the clipping was satisfactory and the parent artery was unobstructed (Fig. 2h, i). Postoperative CT and CTA reexamination revealed no aneurysm development and no obvious hemorrhage in the operative area (P2 in Fig. S1). By following up the patient after discharge, and the patient showed no obvious positive symptoms and had no difference in limb activity compared with the attack.

### Case 3

**Fig. 3:**
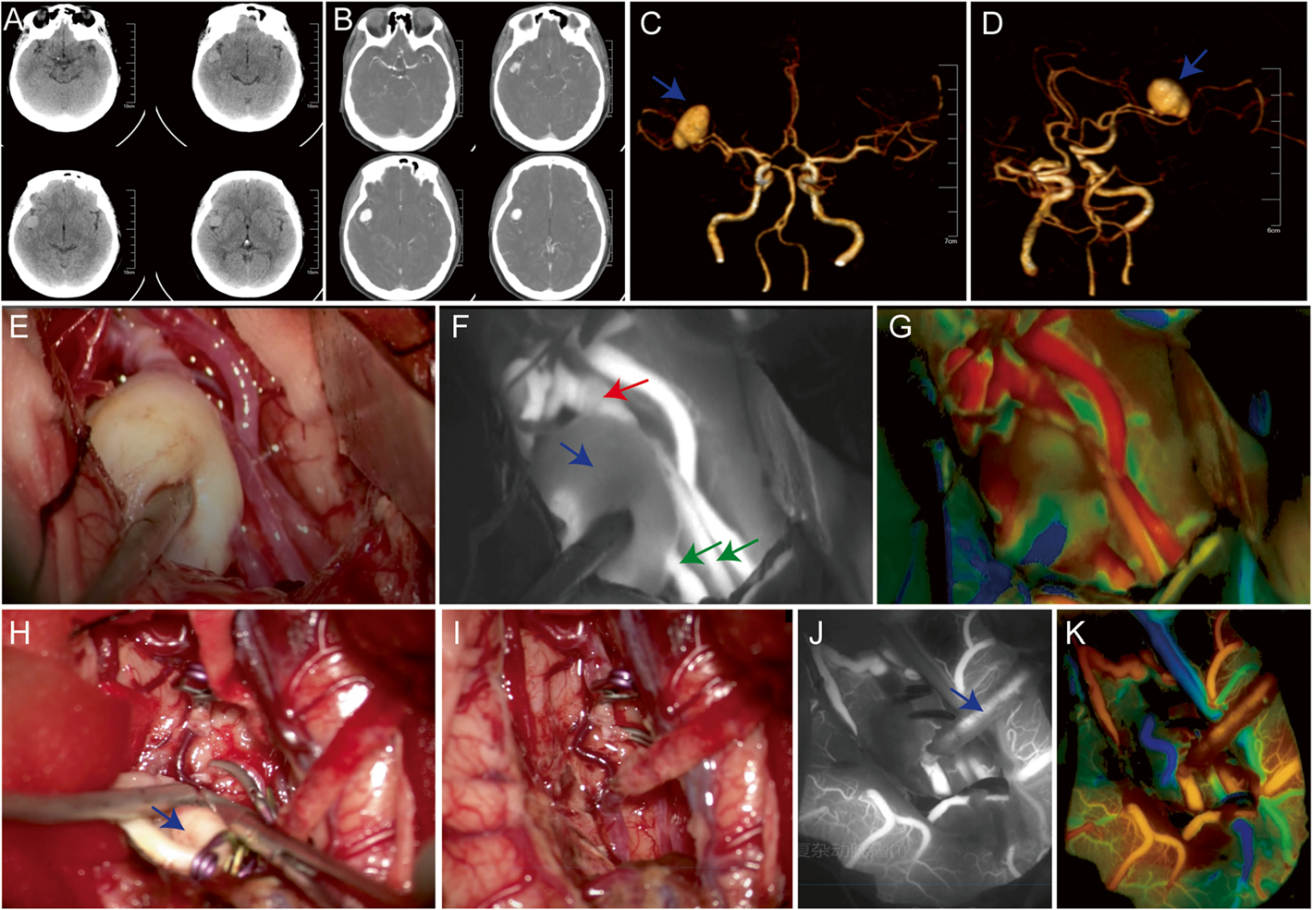
A Right middle cerebral artery (MCA) M2 bifurcation unruptured large aneurysm (20 × 18 mm, blue arrow) with two M3 branches starting from the aneurysm was diagnosed by computed tomography (**a**, **b**) and computed tomography angiography (**c**, **d**). During surgery, we exposed the aneurysm and performed electrocoagulation to shrink it (**e**). Then, conventional indocyanine green video angiography (ICG-VA) showed aneurysm (**f,** blue arrow), parent artery including M2 (red arrow) and two M3 (green arrows). FLOW 800 provided a color-coded map that displayed the brain vessels in contrasting colors depending on the fluorescence appearance after ICG injection (arteries: red, cortical capillaries: yellow/green, veins: blue) (**g**). After low flow extracranial-to-intracranial (EC-IC) bypass (superficial temporal artery-M3), we isolated and resected the aneurysm (**h**, **i,** blue arrow). Another ICG-VA (**j**) and FLOW 800 analysis (**k**) showed the aneurysm disappeared and the bypass vessel was unobstructed (blue arrow)

During routine CT and CTA screen in a 62-year-old male (No. 26 in Table S1), a right MCA aneurysm which was located in the bifurcation of the M2 segment of the right MCA was detected (20 × 18 mm) (Fig. 3a–d). The right frontotemporal arc incision was used intraoperatively. The right M2 and M3 were exposed after separating the side crack. An aneurysm of about 2.0 × 1.8 cm in size at the M2 bifurcation can be clearly seen. Two M3 emanate from the aneurysm. Decided to dissociate the superficial temporal artery because aneurysm is hard to shape and low flow extracranial-to-intracranial (EC-IC) bypass (superficial temporal artery-M3) was adopted. During the surgery, microvascular Doppler exploration revealed obvious blood flow in M3 while high blood flow resistance was showed in M2. Aneurysms were temporarily isolated due to another M3 reflux condition available. Repeated ICG-VA and FLOW 800 suggested unobstructed blood flow of bypass M3, such as the un-bypass M3 had collateral blood supply artery (Fig. 3e–g). After the M3 branch was blocked with aneurysm clip, the aneurysm was removed and ICG-VA was repeated. FLOW 800 suggested that the aneurysm disappeared and the bypass artery had good blood flow (Fig. 3 h–k). TCD re-examination on the first day after the operation showed that the blood flow of the bypass vessel was unobstructed. No infarction and fresh bleeding were seen on postoperative CT scans. CTA showed fluent blood flow of the bypass artery three days later, and the CTP shown that there was no obvious abnormal perfusion in brain tissue after operation (P3 in Fig. S1). After discharge, the patient reported good limb movement and was able to work and live as normal as before the onset of the disease.

## Discussion

In the treament of complex intracranial aneurysms, clipping, bypass, and resection are the main surgical methods. And there are more options for aneurysm clipping in the present clinical work. Microsurgical clipping of cerebral aneurysms is likely to lead to cerebral infarction. According to relevant reports, the average incidence of cerebral infarction after surgical clipping of unruptured aneurysms is 11.3% (10.9% (208/1917) [19], 12.1% (44/363) [20], 12.6% (43/338) [21]), while the average incidence of cerebral infarction after surgical clipping of ruptured aneurysms is 37.9% (21.0% (33/157) [22], 21.0% (33/157) [23]). It can be seen that the incidence of cerebral infarction after clipping ruptured aneurysms is three times as high as unruptured aneurysms, which is related to the imperfect preoperative preparation and the lack of effective intraoperative means to evaluate the hemodynamic changes after aneurysm clipping. Therefore, it is very important to find a way to instruct the surgeon to adjust the intraoperative aneurysm clip and change the surgical method.

ICG is a near infrared fluorescent tricarbonine dye used in medical diagnosis. The dye is injected intravenously, binds tightly to plasma proteins, and is confined to the endovascular system. ICG is eliminated from the bile circulation only by hepatic metabolism with a half-life of 3 to 4 min [24]. ICG-VA can observe the patency of blood vessels, complete clipping of aneurysms, and the blood flow in each branch of blood vessels during the operation. Combination with the high-resolution images of the microscope, it can provide basis for clinicians to timely adjust the surgical plan or aneurysm clip during the operation. Although these merits make ICG-VA look promising, the lack of measurable quantitative values of ICG-VAG raised much concern. It has been reported that the consistency rate between ICG-VA and the intraoperative DSA is only 75% [10], which requires further development of auxiliary tools to help improve the accuracy of ICG-VA. FLOW 800 is an excellent semi-quantitative analysis software. By analyzing the fluorescence intensity after ICG injection and generating color-coded graphs, the direction of blood flow and the patency degree of blood vessels can be determined according to the intensity of the color, especially the effective evaluation of blood vessels at the surgical site and optimized assessment of the vascular anatomy [11]. FLOW 800 technology does not measure the absolute rate of flow but rather provides reliable comparable flow among the vasculature within the field of view as mentioned. Although FLOW 800 has been reported in many literatures in the current surgical operation, the application of FLOW 800 in intracranial complex aneurysms has been rarely reported, especially for the lack of quantitative analysis of flow data during aneurysm surgery. DSA can evaluate the effect of the operation better, but due to its time-consuming and complex operation, the DSA scanning is obviously not realistic in the procedure, so that many hospitals have abandoned the gold standard. When combination of the information generated by ICG-VA and the FLOW 800 generated blood flow perfusion, it can be used as a supplement to provide the blood perfusion of the small arteries, and the surgical procedure before the operation is performed by the surgeon to evaluate the effect of the operation in time, and even to remedy the adverse events of the operation. What is more important is that due to the safety and timeliness of ICG-VA, doctors can perform angiography several times during the operation to continuously improve the operation quality through FLOW 800. By repeating ICG-VA, the stenosis of the parent artery, branch artery, and bypass artery vessels and the velocity of blood flow in the artery after the critical surgical procedure can be determined. In the long run, in combination with the patients with the postoperative CT scan, TCD, DSA, and MR scan, strong guidance can be provided for the treatment rehabilitation training of patients.

However, due to too much subjectivity during the intraoperative application of ICG-VA and FLOW 800, some situations might appear intraoperatively such as blood clots or brain tissue blocking arteries, or arteries are too deep to be observed, which requires strong intraoperative decision-making ability of the surgeon. Meanwhile, there are also problems caused by unhealthy arteries of patients, such as calcification, atherosclerosis, and abnormal thickening of blood vessel walls, resulting in the observed weakening of fluorescence intensity, which also presents a new challenge to the intraoperative judgment of surgeon. It can be seen that although FLOW 800 is a good supplement to ICG-VA, there are still many imperfections to be improved. In brief, although FLOW 800 analysis can provide additional information for ICG-VA to detect hemodynamically related arterial stenosis during surgery, it may further improve the safety of microsurgical clipping. However, since there are few clinical cases available, larger comparative studies are necessary to provide clear conclusions about this approach.

There are some shortcomings in this paper. First, we have not provided the semiquantitative data regarding flow status in aneurysm, major feeding arteries, branching, and bypass vessels compared to ICG-VA. While the data is critical for intraoperative blood flow assessment and postoperative patient health prediction, in the future work, we will attach great importance to the collection and arrangement of relevant data. Secondly, we did not conduct a complete analysis of the postoperative health status of the patients, including complete imaging examination, long-term follow-up investigation, regular review of auxiliary examinations, and other data. In the future, we will establish a sound post-operative health assessment system for patients with complex aneurysms, such as regularly investigate the health status of patients and urging them to make regular re-examinations.

## Conclusion

ICG-VA and FLOW 800 provide high resolution images and they produce the direction and relative magnitude of flow to and from vascular lesions for surgeon, which can be used to evaluate the patency of the parent artery, branch artery, and bypass artery to change the decision during surgery. However, the information is subjective and needs to be judged by surgeons with rich experience. Therefore, the beneficial effects of FLOW800 and aneurysm patient recovery need to be further demonstrated.

## Supplementary Information

**Additional file 1: Table S1**. Details of FLOW 800 findings for all cases with associated operative information. **Figure S1**. The postoperative imaging data of three representative patients.

## Data Availability

Please contact author for data requests.

## Abbreviations

AcomA: Anterior communicating artery
ACA: Anterior cerebral artery
AChA: Anterior choroidal artery
AICA: Anterior inferior cerebellar artery
AVM: Arteriovenous malformation
BA: Basilar artery
CCF: Carotid-cavernous fistula
CT: Computed tomography
DSA: Digital subtraction angiography
ECA: External carotid artery
EC-IC: Extracranial-to-intracranial
GCS: Glasgow Coma Scale
ICA: Internal cerebral artery
ICG: Indocyanine green
LSO: Lateral supraorbital
mRS: Modified Rankin score
MCA: Middle cerebral artery
MRI: Magnetic resonance imaging
PA: Pericallosal artery
PCA: Posterior cerebral artery
PcomA: Posterior communicating artery
PICA: Posterior inferior cerebellar artery
RIA: Ruptured intracranial aneurysm
ROI: Region of interest
TCD: Transcranial doppler
SAH: Subarachnoid hemorrhage
SCA: Superior cerebellar artery
SD: Standard deviation
STA: Superficial temporal artery
UIA: Unruptured intracranial aneurysm
VA: Videoangiography
