# Supplementary material for: Intraoperative indocyanine green video angiography (ICG–VA) with FLOW 800 software in complex intracranial aneurysm surgery
Source: Chin Neurosurg J. 2021 Jun 1;7:28. doi: 10.1186/s41016-021-00247-z (PMC8168317; doi:10.1186/s41016-021-00247-z)

Intraoperative indocyanine green video angiography (ICG–VA) with FLOW 800 software in complex intracranial aneurysm surgery

Tao Xue[1,#], Ruming Deng[2,#], Bixi Gao[1], Zilan Wang[1], Chao Ma[1], Wanchun You[1], Yun Zhu[1], Zhouqing Chen[1,*], Zhong Wang[1,*]

[1] *Department of Neurosurgery & Brain and Nerve Research Laboratory, The First Affiliated Hospital of Soochow University, Suzhou, Jiangsu Province, 215006, China*

[2] *Department of Neurosurgery, Bozhou People's Hospital, Anhui Province, China*

Complete co-author email addresses:

Tao Xue, MD, E-mail: 2992326676@qq.com

Ruming Deng, MD, E-mail: 49510739@qq.com

Bixi Gao, MD, E-mail: gaobixi140508@163.com

Zilan Wang, MD, E-mail: zlwang@stu.suda.edu.cn

Chao Ma, MD, E-mail: machaoss@163.com

Wanchun You, MD, E-mail: wcyou@suda.edu.cn

Yun Zhu, MD, E-mail: zy7235zy@163.com

Zhouqing Chen, MD, E-mail: zqchen6@163.com

Zhong Wang, MD, E-mail: wangzhong761@163.com

[#] Tao Xue and Ruming Deng contribute equally to this work.

*Corresponding author: Zhouqing Chen or Zhong Wang Department of Neurosurgery, The First Affiliated Hospital of Soochow University, 188 Shizi Street, Suzhou 215006, China. E-mail address: wangzhong761@163.com or zqchen6@163.com

Keywords: aneurysm; bypass; clipping; FLOW 800; ICG-VA

Short title: FLOW 800 analysis for aneurysm treatment.

No.	Pathology	Operation	Complexity of aneurysms, intraoperative strategy, and Flow 800 findings
1	Left ICA C5 paraclinoid aneurysm	Clipping	The aneurysm was located under the anterior clinoid and we grinded the bone of the anterior clinoid carefully to expose the aneurysm. The diameter of the aneurysm is about 1.1cm. A cross-window clip was applied because the aneurysm points to the inside. FLOW 800 analysis confirmed complete occlusion of aneurysm and adequate flow in parent artery.
2	Left ICA C6 large recurrent aneurysm	Trapping + high flow EC-IC bypass	The aneurysm was 16.6*12.3 mm in size and had previous treatment. We trapped the aneurysm at left ICA initial part and ICA before PComA branch after high flow EC-IC bypass (M3-RA-superior thyroid artery). FLOW 800 analysis indicated the bypass vascular was fluent and peripheral blood flow improved.
3	Left ICA C4 giant aneurysm with CCF	Trapping + high flow EC-IC bypass	The aneurysm was 25*20 mm in size and the patient also had left CCF. We performed high flow EC-IC bypass (M2-RA-ICA). Then, we trapped aneurysm at ICA initial part and ICA before PComA branching. FLOW 800 analysis and intraoperative ultrasound showed the aneurysm disappeared and the bypass vascular was fluent.
4	Left MCA giant ruptured aneurysm	Trapping + high flow EC-IC bypass	The aneurysm was ruptured with much bleeding and 25*15 mm in size. We first trapped aneurysm at proximal MCA and distal MCA to control bleeding. Then we excised the aneurysm and performed high flow EC-IC bypass (M2-RA-ECA). FLOW 800 analysis and intraoperative ultrasound confirmed the bypass vascular was fluent. Afterwards, distal MCA was released and FLOW 800 was used again to confirm the bypass vascular was unobstructed.
5	Left ICA C4-C6 giant aneurysm	Trapping + high flow EC-IC bypass	The aneurysm was 40*22 mm in size. high flow EC-IC bypass (M3-RA-superior thyroid artery) was applied to this patient before the aneurysm was trapped. FLOW 800 analysis exhibited the bypass vessel was fluent.
6	Left PcomA giant aneurysm	Clipping	The aneurysm was about 24 mm in diameter. Combination of two clips (762, 782) was used to mold and clip the giant aneurysm. The first FLOW 800 detected the aneurysm was clipped completely but the parent artery was stenosis. Then, we repositioned the second clip and FLOW 800 and intraoperative TCD both indicated the blood flow of parent artery recovered.
7	AcomA aneurysm	Aneurysmectomy	The aneurysm (23*15 mm in size) had intraluminal thrombus and calcification of the aneurysmal wall. First, we separated the aneurysm neck and ipsilateral A2, temporary blocked ipsilateral A1, A2, positioned an aneurysm clip, loosened the temporary block, FLOW 800 indicated A2 without blood flow, debugged aneurysm clip, A2 still had no blood flow. Then, ipsilateral A1 and A2 were blocked again, the aneurysm was cut open, intra-aneurysm thrombus was removed, and aneurysm was resected. Contralateral A1, anterior communicating artery and A2 were seen below. The aneurysm neck was thin and was not able to be clipped. Therefore, we tried to suture anterior traffic artery and ipsilateral A2 blood vessel. After suture, release temporary block, blood flow was acceptable. However, oozing blood was found, and suture was added. We removed residual aneurysm, detected ipsilateral A2 again and found no blood flow signal by FLOW 800. Considering intravascular thrombosis, we dismantled anastomosis, removed the fresh blood clots, used heparin saline flushing repeatedly, saw the endangium injury and sutured blood vessels again. Afterwards, we opened suture again, still saw a thrombosis, loosened the A2 temporary

			block, saw the retrograde bleeding obvious, considered the remote vessels well compensated, and hence gave up blood vessel suture, Finally, we closed ipsilateral A1, A2 and AcomA.
8	Left VA V4-BA giant dissecting + left PCA P2 fusiform + left PComA + bilateral ICA C1 multiple aneurysms	Clipping + high flow EC-IC bypass	We clipped the left PComA aneurysm (12*6 mm in size) with a 644 cross-window right-angle clip. FLOW 800 analysis showed the parent artery was fluent. Then, we performed high flow EC-IC bypass (PCA-RA-superior thyroid artery) and FLOW 800 analysis indicated the bypass vessel was unobstructed.<br>The patient's condition was extremely complex. Multiple aneurysms were not ruptured, but the vertebrobasilar dissection aneurysm was huge with intraluminal thrombosis. The patient also had obvious neurological defect like posterior circulation ischemia and cranial nerve damage. One operation was difficult to deal with multiple intracranial aneurysms. After discussion in the department, the left ECA-RA-PCA bypass plus left PComA aneurysm clipping were performed, and the left VA V4-BA giant dissecting aneurysm was occluded by intravascular balloon. Left PCA aneurysm was difficult to deal with because its shape (fusiform) and bilateral ICA C1 aneurysms were not handled at this time.
9	Right ruptured MCA + right AcomA + left ICA C6 multiple aneurysms	Clipping	We prepared to deal with the right ruptured MCA aneurysm (6*4 mm in size) and right AcomA aneurysm (5*2 mm in size) through right pterional approach. Firstly, the right MCA aneurysm was clipped satisfactorily with a 940-aneurysm clip and the branches of the MCA were satisfactorily preserved, which were confirmed by FLOW 800 analysis. Then, the A1 segments of the bilateral ACAs were exposed and temporarily blocked. We separated the right ACA aneurysm neck and positioned a 742-aneurysm clip. However, it was found that the clipping was not complete, and the 742-aneurysm clip was replaced by a 940-aneurysm clip. The residual in the aneurysm neck was strengthened with two mini clips. Flow 800 indicated the aneurysm was clipped completely, and the bilateral A1 and A2 vessels were unobstructed.
10	Right PICA aneurysm	Clipping	The aneurysm (2.3*2 mm in size) was exposed after we separated along the proximal to the distal end of right PICA. Then, the aneurysm was clipped and the parent artery was preserved, which were verified by Flow 800 analysis.
11	Right PcomA + AChA multiple aneurysms	Clipping	A saccular aneurysm (3*3 mm in size) could be seen at about 5mm from the bifurcation of right ICA, pointing to the outside, and the AChA went out at the proximal of the aneurysm. Further exploration to the proximal of ICA showed that there was a lobulated aneurysm at the dorsal side of right PcomA, pointing to the posterior inferior and posterior lateral side respectively. The, we separated the PcomA and its surrounding branches. The PcomA aneurysm was clipped by a transvascular T-Bar-shaped aneurysm clip satisfactorily, and then the AChA aneurysm (8*4 mm in size) was clipped satisfactorily by a 940-aneurysm clip. Flow 800 analysis revealed that no parent artery stenosis was found in both places, and the branch vessels were well preserved.
12	BA apex aneurysm	Clipping	The right lateral supraorbital (LSO) approach was used to open the second space and the superior space of the internal carotid artery. Then we cut the liliequis membrane, exposed the posterior clinoid process, and showed the BA apex aneurysm, pointing upward, about the size of 4mm. After that we separated the aneurysm neck and clipped the aneurysm with one 940-aneurysm clip (Aesculap, Germany) satisfactorily. Finally, FLOW 800 analysis indicated complete occlusion of

			the aneurysm and sufficient flow in parent artery.
13	AICA distal ruptured aneurysm	Clipping + trapping + aneurysmectomy	There was a small amount of old hematoma on the left lateral side of the medulla. After removal, the aneurysm (5*3 mm in size) with high tension, adhesion with the posterior cranial nerve, was carefully separated. The parent artery was thin. The aneurysm ruptured again during separation and the parent artery was blocked. Eventually, the aneurysm was trapped and resected. FLOW 800 analysis demonstrated the aneurysm disappeared.
14	Left PcomA aneurysm with intraluminal thrombus	Clipping	During the separation, the aneurysm (5.4*3.8 mm in size) ruptured and the proximal ICA was temporarily blocked. We clipped the aneurysm with 752 (Aesculap, Germany) aneurysm clip. Intraluminal thrombus was seen during the operation. After adjusting the aneurysm clip slightly, the ICA, PComA and AChA were examined for distortion. FLOW 800 analysis was performed to confirm the blood flow was unobstructed, and no residual of aneurysm was found.
15	Left ICA-ACA-AcomA giant aneurysm with intraluminal thrombus	Clipping + aneurysmectomy + low flow EC-IC bypass	Firstly, the end-to-side anastomosis between the parietal branch of STA and M4 segment of MCA was performed under microscope. TCD monitoring showed that the anastomosed vessels were unobstructed and the pulse was good. Then, we separated the lateral fissure and the arachnoid of the skull base, lifted the temporal lobe, and found the giant aneurysm (54*45mm in size). ICA in the neck and distal end of the parent artery were temporarily blocked. Afterwards, the aneurysm was cut open, thrombus and organized tissue were removed, and most of the aneurysm was resected. Two aneurysm clips were positioned along the aneurysm neck and then we loosened the temporary blocking. The intraoperative electrophysiological monitoring showed nothing abnormal was detected. Intraoperative TCD and FLOW 800 analysis confirmed that the blood flow of the parent artery and bypass vessel was unobstructed. The giant aneurysm disappeared.
16	Right SCA aneurysm	Clipping	When the second space was opened, trunk of the basilar artery could be seen on the deep side. After further separation and enlargement of the space, it could be seen that the aneurysm (2.5*2.3 mm in size) was located at the intersection of the BA and the right SCA. Then, we separated the aneurysm neck carefully, and clipped the aneurysm satisfactorily by a 940 clip. There was no stenosis in the BA and SCA. FLOW 800 analysis indicated that the aneurysm was completely occlusive and the blood flow of BA, SCA and PCA was fluent.
17	Right MCA multiple large aneurysms	Clipping + aneurysmectomy + trapping + low flow EC-IC bypass	Two aneurysms were 18.10*14.74mm and 18.02*13.87mm in size respectively. After the M1 segment of right MCA was separated, one of the aneurysms was seen, which originated from the bifurcation of the temporal polar artery. Careful separation of the aneurysm showed that there was hyperplasia and calcification of the aneurysm wall. Then, we temporarily blocked the beginning of M1, and found obvious thickening of the aneurysm wall after incision of the aneurysm. After removing most of the aneurysm, we tried to clip the aneurysm with the combination of right-angle clips, cross-vessel clips, and other kinds of aneurysm clips. FLOW 800 analysis found that the blood flow signal in the parent artery was weak and repeated adjustment of the aneurysm clips failed to maintain the patency of the parent artery. The other aneurysm was located at the beginning of the temporal pole artery, and the temporal polar artery was located on the aneurysm. The aneurysm was trapped directly. Afterwards, low flow EC-IC bypass (STA-M4) was performed by end-to-side anastomosis. Intraoperative FLOW 800 analysis confirmed that the anastomosis was unobstructed.

18	Right ICA C6 aneurysm with sellar mass	Clipping + tumor resection	There was a cystic lesion in the first space. The tumor capsule wall was cut after electrocoagulation and there was some yellowish liquid in it. The whole cyst wall was removed, and the normal pituitary gland and pituitary stalk were well protected. Then, we gently lifted the frontal lobe, saw the ICA, optic nerve, and carefully separated the arachnoid around ICA. The aneurysm (10.5*7.2 mm in size) was located under the anterior clinoid process. We carefully grinded the anterior clinoid to and exposed the proximal neck of the aneurysm. Afterwards, the aneurysm neck was separated and clipped with 640T, 854T and 644T (Aesculap, Germany) aneurysm clips. FLOW 800 exhibited that the aneurysm was clipped satisfactorily and there was no stenosis of the parent artery.
19	Left ICA C6 large aneurysm	Clipping + low flow EC-IC bypass	At first, the aneurysm (22.5*20 mm in size) was electrocoagulated and shrunk slightly. Then, it was clipped with two cross-window right-angle aneurysm clips (654), and there was still a small amount of bleeding. Afterwards, a 786 great curved aneurysm clips were used to strengthen the aneurysm. Intraoperative TCD monitoring and FLOW 800 analysis showed that the aneurysm was clipped completely and the blood flow was unobstructed by ICA, MCA, ACA. Finally, low flow EC-IC bypass (STA-M4) was performed and the second time FLOW 800 analysis confirmed that the bypass vessel was fluent.
20	Left PCA ruptured aneurysm, left PComA aneurysm?	Clipping	The initial part of the PComA was cystic dilated, and PComA was slender, starting from the wall of the cystic dilated vessel. There was adherent organized hematoma under the optic chiasma. After partial removal of the hematoma, it could be seen that one aneurysm (1.6*1.5 mm in size) was located at the beginning of the left PCA. The aneurysm neck was carefully separated and the aneurysm neck was exposed satisfactorily. A 742-aneurysm clip was used, and there was a small amount of bleeding in the process of clipping. A small amount of residual aneurysm was found in the distal end of the aneurysm. A 940 clip was applied. FLOW 800 analysis showed satisfactory clipping of the aneurysm and no stenosis of the parent artery. After consideration, the initial part of the PComA was dilated obviously, and the arterial wall was weak under the microscope. A mini clip was used to clamp the enlarged protuberant vessels.
21	Left ICA C6 aneurysm	Clipping	We grinded the bone of the anterior clinoid carefully to expose the aneurysm (20.5*12.7 mm in size). A cross-window clip was used to clamp the aneurysm. FLOW 800 analysis confirmed the blood flow of parent artery was unobstructed.
22	Left PComA aneurysm	Clipping	The aneurysm (10.3*8.9 mm in size) was located outside of the left ICA, pointing outward and downward, with obvious peripheral adhesion. The PComA went out from the proximal aneurysm neck. We dissociated the aneurysm neck and clipped the aneurysm neck with a cross-window aneurysm clip. Intraoperative FLOW 800 analysis indicated that the clipping was satisfactory and the parent artery was unobstructed.
23	BA apex and right MCA M2 multiple aneurysms	Clipping	First, the BA apex aneurysm (2.86*3.11 mm in size) was clipped by a 752-aneurysm clip and FLOW 800 analysis showed the aneurysm was clipped satisfactorily and bilateral PCAs, SCAs and other surrounding vessels were fluent. Then, a mini clip was applied to deal with the M2 aneurysm (4.56*2.77 mm) and FLOW 800 analysis showed the aneurysm was also clipped satisfactorily and the parent artery was unobstructed.

24	Left PComA aneurysm with calcification of wall	Clipping	We took the incision of the left LSO approach. The aneurysm was located at the anterior skull base and the diameter of the aneurysm is about 0.9cm, it was difficult to expose the aneurysm. We carefully detected that the aneurysm neck was located outside of the anterior clinoid process, and the aneurysm wall was calcified. After temporary blocking of ICA, the aneurysm was clipped with a 960-aneurysm clip, and then a straight aneurysm clip was given to strengthen the aneurysm. The aneurysm clipping was satisfactory. TCD and FLOW 800 analysis confirmed that the parent artery was unobstructed during the operation.
25	AComA ruptured aneurysm with previous treatment	Clipping	The patient underwent clipping of AComA aneurysm (6.7*4.9 mm in size) 3 months ago. After exposing the ipsilateral A1, A2 and contralateral A1, A2, it was found that the original aneurysm was clipped well, and another AComA aneurysm ruptured. A straight aneurysm clip was used to clamp the aneurysm, and the aneurysm was satisfactorily clipped. No obvious vascular distortion was found in FLOW 800 analysis, and the blood flow of the ipsilateral A1, A2 and contralateral A1, A2 was unobstructed, and the aneurysm was not developed.
26	Right MCA M2 bifurcation large aneurysm	Aneurysmectomy + trapping + low flow EC-IC bypass	The aneurysm was about 20*18 mm in size, and two M3 branches emanated from the aneurysm. It was very difficult to shape and clip the aneurysm. Therefore, low flow EC-IC bypass (STA-M3) was performed and the blood flow was unobstructed confirmed by FLOW 800. The aneurysm was isolated and resected. Finally, another intraoperative FLOW 800 indicated that M3 had blood flow, and bypass vessel was unobstructed.
27	Left MCA bifurcation ruptured broad-base aneurysm	Clipping + low flow EC-IC bypass	The aneurysm (10.7*9.8 mm in size) was seen at the bifurcation of M1 segment of MCA, and the tension of aneurysm was high. We separated aneurysm with two branches M2 and found the aneurysm neck was wide. One M2 segment was involved, and the wall was thin, so conventional clipping was very difficult. We first performed low flow EC-IC bypass (STA-M2). TCD monitoring showed that the anastomotic vessels were unobstructed and the pulse was good. Then, the aneurysm was reshaped to ensure the blood supply of another M2. Seven aneurysm clips (950T*4, 710T*2, 960T*1) were used to reshape the aneurysm neck. Under the monitoring of TCD during operation, the positions of aneurysm clips were adjusted repeatedly to ensure that the blood flow of M1 segment and M2 segment was smooth, and FLOW 800 analysis was conducted to confirm the patency of blood flow. However, the blood flow of bypass vessel (STA-M2) was not detected again, and FLOW 800 analysis also indicated that the blood flow was blocked. Considering the anastomotic thrombosis, the anastomosis was opened, the fresh thrombus was removed, and the vessels were washed with heparin saline repeatedly. The vascular intima was intact, so the end-to-side anastomosis was performed between the frontal branch of STA and the original anastomosis. Finally, intraoperative FLOW 800 analysis and TCD exhibited the blood flow was unobstructed.
28	Right MCA M3 large aneurysm	Aneurysmectomy + trapping + low flow EC-IC bypass	The aneurysm (15*12 mm in size) was in the M3 segment of the right MCA. We blocked M2 and M3 segments and resected the aneurysm. The STA was sutured end-to-end to the MCA-M3. Intraoperative TCD and FLOW 800 showed that the anastomotic vessels were unobstructed and the pulse was good.
29	Right	Clipping	The aneurysm (20*15 mm in size) was in the M1 bifurcation of the right MCA. At first, the neck of

	ruptured MCA bifurcation large aneurysm		the aneurysm was clipped with a 950-aneurysm clip, but the neck was too wide to be completely clipped. Then, a 960-aneurysm clip was added to the lateral side, but it was still not completely clipped. We added another 742 and 786 aneurysm clips, adjusted clips repeatedly, and clipped the aneurysms. FLOW 800 analysis indicated clipping of the aneurysm was satisfactory, the blood flow of M1, M2 was unobstructed, and no obvious distortion of blood vessels was detected.
30	Left ICA C6 aneurysm	Clipping	We grinded the bone carefully to expose the aneurysm (3.7*2.3 mm in size), which was located inside of the anterior clinoid process. We temporarily blocked the ICA and clipped the aneurysm with a cross-window right-angle clip. Intraoperative FLOW 800 analysis confirmed the clipping of the aneurysm was satisfactory and the parent artery was unobstructed.
31	BA ruptured aneurysm	Clipping	The aneurysm (8.1*5.2 mm in size) was clipped with a 940-aneurysm clip. FLOW 800 analysis showed the clipping of the aneurysm was satisfactory and the BA and bilateral PCAs were unobstructed.
32	Left AICA ruptured aneurysm	Clipping	Under the microscope, the left VII, VIII nerves were exposed, and we found the left AICA went through the VII, VIII nerves. The aneurysm (5.92*2.72 mm in size) was seen on the ventral side of VIII nerve. We carefully separated the aneurysm and clipped it with a 740-aneurysm clip. The important structures such as facial nerve, acoustic nerve, trigeminal nerve, posterior cranial nerves, brainstem were and the petrosal vein were well preserved. Intraoperative FLOW 800 analysis showed that the parent artery was unobstructed and there was no stenosis.

Table S1. Details of FLOW 800 findings for all cases with associated operative information

ICA = internal carotid artery; high flow EC-IC = extracranial-to-intracranial; PComA = posterior communicating artery; AComA = anterior communicating artery; CCF = carotid-cavernous fistula; MCA = middle cerebral artery; ECA = external carotid artery; ICG-VA = Indocyanine green video angiography; VA = vertebral artery; PICA = posterior inferior cerebellar artery; AVM = arteriovenous malformation; ACA = anterior cerebral artery; AChA = anterior choroidal artery; BA = basilar artery; LSO = lateral supraorbital; AICA = anterior inferior cerebellar artery; STA = superficial temporal artery; SCA = superior cerebellar artery; PCA = posterior cerebral artery; TCD = transcranial doppler.

Figure S1. The postoperative imaging data of three representative patients

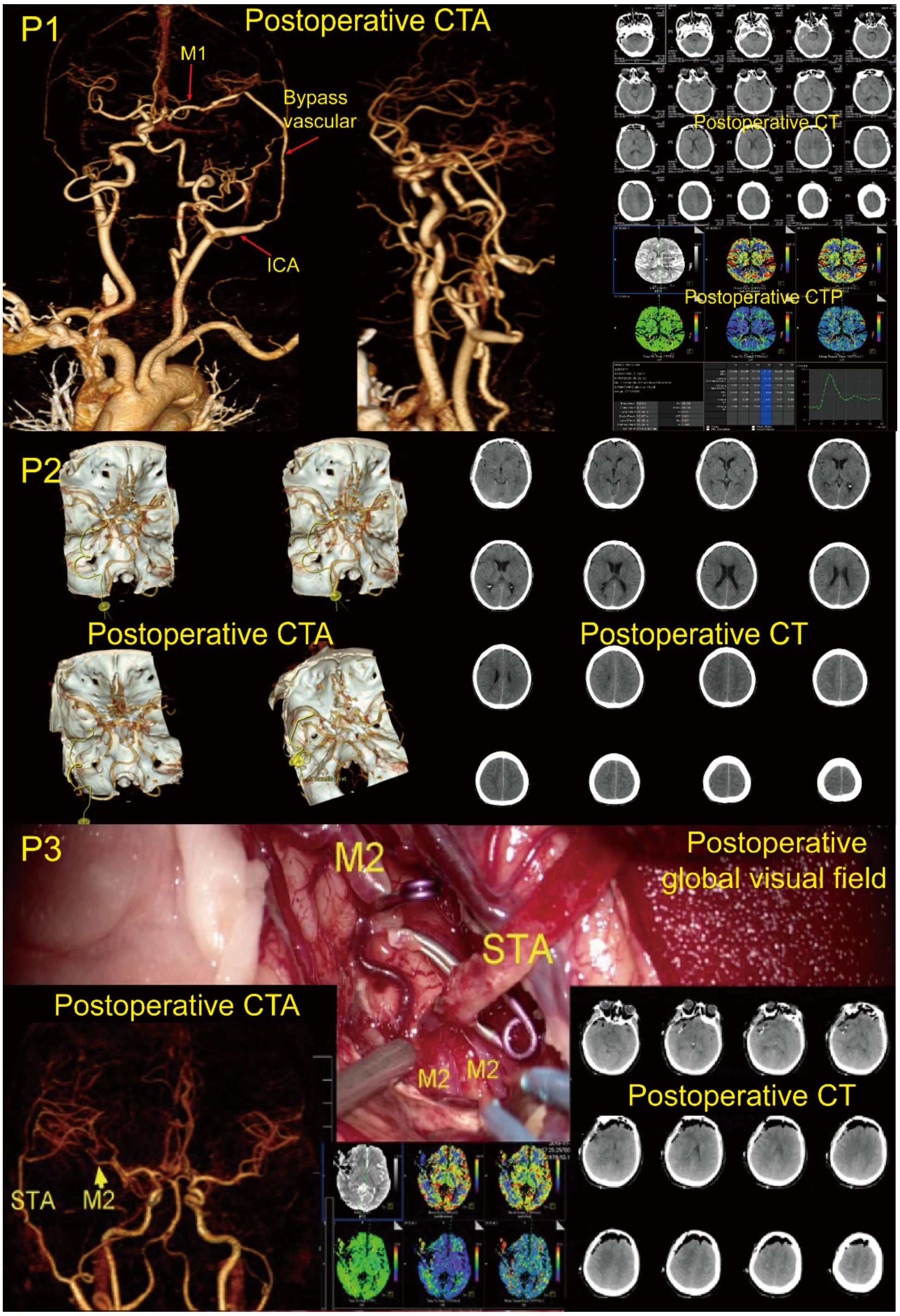

Supplement: Supplementary file 1 — Additional file 1: Table S1. Details of FLOW 800 findings for all cases with associated operative information. Figure S1. The postoperative imaging data of three representative patients. [file 41016_2021_247_MOESM1_ESM.pdf]
